# Coexistence of carbapenemase and hypervirulence-associated genes among *Klebsiella pneumoniae* high-risk clones in Hungary

**DOI:** 10.3389/fmicb.2026.1870558

**Published:** 2026-08-19

**Authors:** Lilla Buzgó, Csongor Freytag, Dániel Göbhardter, Levente Laczkó, László Miló, Leila Holub, Adrienn Hanczvikkel, Erika Ungvári, László Majoros, Katalin Kamotsay, Katalin Papp, Katalin Kristóf, Gábor Kardos, Ákos Tóth

**Affiliations:** 1 Department of Bacteriology, Parasitology and Mycology, National Center for Public Health and Pharmacy, Budapest, Hungary; 2 Department of Infection Control and Hospital Epidemiology, One Health Institute, Faculty of Health Sciences, University of Debrecen, Debrecen, Hungary; 3 Department of Bioinformatics, One Health Institute, Faculty of Health Sciences, University of Debrecen, Debrecen, Hungary; 4 HUN-REN-UD Conservation Biology Research Group, University of Debrecen, Debrecen, Hungary; 5 Centre for Metagenomics, University of Debrecen, Debrecen, Hungary; 6 Department of Socio-Geography and Regional Development, Institute of Earth Sciences, Faculty of Science and Technology, University of Debrecen, Debrecen, Hungary; 7 University of Debrecen Clinical Centre, Faculty of Medicine, Institute of Medical Microbiology, Debrecen, Hungary; 8 South Pest Central Hospital National Institute of Hematology and Infectology, Microbiology Laboratory, Budapest, Hungary; 9 Szabolcs-Szatmár-Bereg County Hospital and University Teaching Hospital, Central Laboratory, Nyíregyháza, Hungary; 10 Semmelweis University, Faculty of Medicine, Laboratory Medicine Institute, Budapest, Hungary; 11 Department of Planetary Health, One Health Institute, Faculty of Health Sciences, University of Debrecen, Debrecen, Hungary

**Keywords:** blaNDM-1 carbapenemase, hybrid plasmids, hypervirulence-associated genes, *Klebsiella pneumoniae*, plasmid analysis, ST147, whole-genome sequencing

## Abstract

**Introduction:**

Strains of *Klebsiella pneumoniae* carrying hypervirulence and carbapenemase genes represent a rapidly emerging global public health threat. Our study aimed to comprehensively characterise the genomics of hypervirulence-associated and carbapenemase genes carrying *K. pneumoniae* (hv(a)CpKp) isolates in Hungary.

**Materials and methods:**

Between January 2022 and April 2024, 89 aerobactin (*iucA-D*/*iutA*)-positive non-duplicate carbapenemase-producing *K. pneumoniae* isolates from 15 Hungarian healthcare institutes underwent short-read (Illumina, MiSeq, NextSeq) whole-genome sequencing, followed by detailed plasmid analysis using long-read sequencing (Nanopore, MinION) in a representative subset of 32 strains.

**Results:**

Most isolates (79/89) belonged to the high-risk clone ST147. Hypervirulence-associated (hva) genes—including *rmpA/rmpA2, peg344, shiF, iucA–D*, and *iutA*—were universally present, and 59 isolates possessed chromosomally integrated yersiniabactin loci. Most isolates (87/89) carried the *bla*_NDM-1_ carbapenemase gene. Hypervirulence-associated genes were most frequently (29/32) associated with IncHI1B/IncFIB(Mar) plasmids. Notably, we identified plasmids carrying both hva and carbapenemase genes—designated as hybrid plasmids—in 13 of 32 strains. The *bla*_NDM-1_ was linked to the *IS26* transposase and was present in conserved, identical cassettes on all *bla*_NDM-1_-carrying plasmids.

**Discussion/conclusion:**

Our study identified hv(a)CpKp strains, particularly the ST147 clone, circulating in Hungary. Our findings highlight the need for routine virulence gene monitoring and continuous genomic and plasmid-based surveillance to mitigate the clinical and epidemiological impact of emerging hv(a)CpKp lineages.

## 1 Introduction

*Klebsiella pneumoniae* is one of the most important pathogen among *Enterobacterales*, which are responsible for a significant proportion of healthcare-associated infections worldwide (Santajit and Indrawattana, 2016; Arena et al., 2022; Abdelsalam et al., 2024). Its global epidemiological success is largely determined by two distinct evolutionary pathways: hypervirulence and multidrug resistance associated with the production of extended-spectrum *β*-lactamases (ESBLs) and/or carbapenemases (Wyres et al., 2020a; Arcari and Carattoli, 2023).

The hypervirulent pathotype was first described in 1986 (Chen et al., 2023), and the infections it caused were mainly community acquired (European Centre for Disease Prevention and Control [ECDC], 2025). Since then, hypervirulent *Klebsiella pneumoniae* (hvKp) strains causing invasive infections have been increasingly reported worldwide (Raro et al., 2023). These strains are often associated with severe clinical manifestations—even in healthy individuals—such as liver abscesses, endophthalmitis, and meningitis (Zhu et al., 2021; Chen et al., 2023).

Hypervirulence is mainly driven by increased production of capsular polysaccharides (usually mediated by the *rmpA* and/or *rmpA2* genes)—which develop the hypermucoviscous phenotype—and the expression of various siderophores, including aerobactin, enterobactin, salmochelin, and yersiniabactin. Of these, aerobactin is considered the primary virulence determinant and contributes significantly to the pathogenicity of hvKp. Several other genes are also associated with hypervirulence, including *peg344*, a metabolite transporter which is commonly associated with pneumonia, *luxR*, a transcriptional regulator, and *shiF*, involved in lysine transport during aerobactin biosynthesis. In addition, colibactin, a peptide–polyketide genotoxin that promotes bacterial colonisation and impairs host immune responses (Ye et al., 2016; Bulger et al., 2017; Russo and Marr, 2019; Shoja et al., 2022; Mendes et al., 2023). Fimbriae, including type 1 and type 3, also represent important virulence factors that facilitate the adherence to both biotic and abiotic surfaces (Russo and Marr, 2019; Han et al., 2022; Mendes et al., 2023).

The classical *K. pneumoniae*, the other global pathotype, is frequently associated with multidrug resistance (MDR), including carbapenemase-producing strains (CpKp; Russo and Marr, 2019; Zhu et al., 2021; Raro et al., 2023). The resistance mechanism is mainly mediated by various genes for antibiotic-hydrolysing and antibiotic-modifying enzymes located on the chromosome or mobile genetic elements (Han et al., 2022). These include Ambler class A ESBLs (such as CTX-M, SHV, and TEM) and KPC-type carbapenemases, class B metallo-*β*-lactamases (such as VIM and NDM), class C AmpC enzymes (such as DHA and CMY), and class D oxacillinases or carbapenemases (Zhu et al., 2021; Mendes et al., 2023).

In past decades, common hvKp clones such as ST23, ST86, and ST65 and common multidrug-resistant *K. pneumoniae* (MDR-Kp) clones such as ST258, ST147, and ST307 have been clearly distinguished. Recently, however, hypervirulent and multidrug-resistant strains have been increasingly reported, such as the KPC-producing, hypervirulent ST11 and several hypervirulent, carbapenemase-producing ST23 strains, which are emerging in Europe (Wyres et al., 2019, 2020a; Mendes et al., 2023; Raro et al., 2023; ECDC, 2024).

This evolutionary process contributes to the emergence and spread of multidrug-resistant, hypervirulent *K. pneumoniae* (MDR-hvKp) convergent clones (Raro et al., 2023; Abdelsalam et al., 2024). A further concern is that *K. pneumoniae* could probably act as a ‘pool’ again, similar to the ESBL era, this time via plasmids carrying both multidrug resistance genes and hypervirulence genes (Tzouvelekis et al., 2012; Choby et al., 2020; Morgado et al., 2022).

Infections caused by hvKp occur geographically worldwide, e.g., in North and South America, Africa, East and Southeast Asia, and Oceania—the highest prevalence in the Asia-Pacific region but also pose a growing threat in Europe. In addition, the prevalence of carbapenem-resistant hvKp (CR-hvKp) infections has steadily increased since they were first detected in 2015 (Zhu et al., 2021; Chen et al., 2023; Raro et al., 2023). A comprehensive study from China found that 36% of carbapenem-resistant *K. pneumoniae* strains detected also had hypervirulence factors (Liu et al., 2022). While the MDR-hvKP population is heterogeneous worldwide, the most common sequence types (ST) include the KPC-producing ST11 and the dominant hypervirulent lineage ST23 (Raro et al., 2023).

Our study focused primarily on detection of biomarker genes, whose role in the development of the hypervirulent phenotype has also been confirmed *in vivo* infection models. These biomarkers include the aerobactin, salmochelin, and yersiniabactin genes, the *peg344* gene, as well as the plasmid-associated *rmpA* and *rmpA2* genes (Russo et al., 2018; Wyres et al., 2020b; Arcari and Carattoli, 2023; Mai et al., 2023; Rao and Fernandes, 2025). Because the presence of these genes alone does not definitively establish a hypervirulent phenotype, isolates carrying these determinants are referred to throughout this manuscript as hypervirulence-associated and carbapenemase genes carrying *K. pneumoniae* hv(a)CpKp.

The aim of our study was to assess the presence of hypervirulence-associated genes in NDM-producing *K. pneumoniae* strains isolated from Hungarian healthcare institutions (HCIs) and to explore the prevalence and possible convergence of plasmids and clonal lineages carrying both hypervirulence-associated and carbapenemase genes.

## 2 Materials and methods

### 2.1 Study design and bacterial isolates

**Figure 1:**
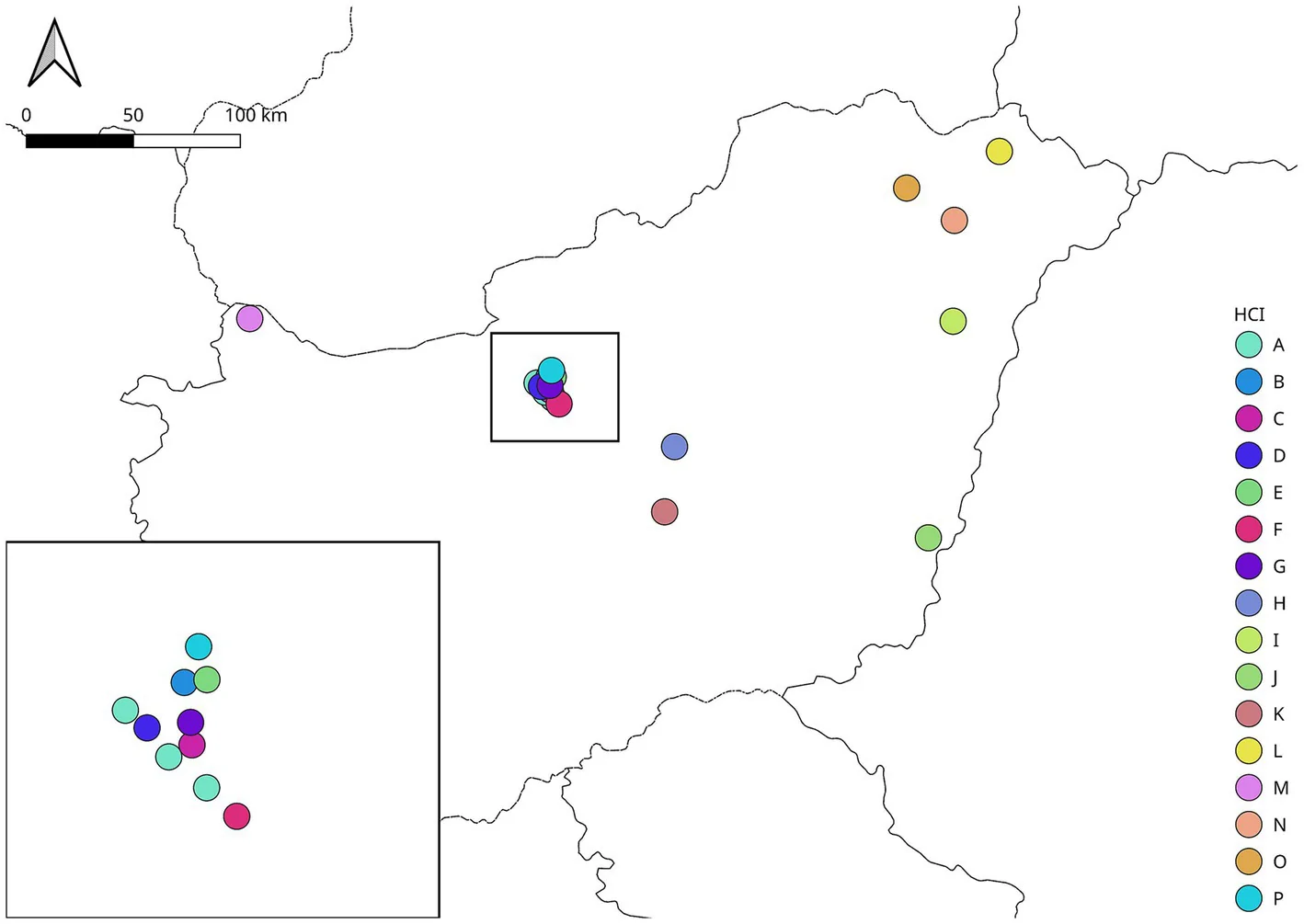
Geographical distribution of HCIs that submitted the studied isolates. Each colour represents a different HCI. The inset shows the HCIs of the capital.

Between January 2022 and April 2024, 540 carbapenemase-producing *Klebsiella pneumoniae* isolates were submitted to the National Reference Laboratory for Antimicrobial Resistance for genomic epidemiological surveillance, as required by Hungarian legislation. In accordance with the rules of procedure, all isolates (*n* = 540) underwent short-read sequencing following confirmation of carbapenemase production. Based on sequencing results analysed using SeqSphere (Ridom, Münster, Germany) and the presence of aerobactin genes (*iucA–D*, *iutA*), 89 non-duplicate isolates from 15 healthcare institutes (HCIs) were selected (Figure 1). In addition, the selected isolates had a categorical virulence score of ≥3, as determined by the Kleborate pipeline via the PathogenWatch platform, which screens for five key virulence loci commonly associated with hvaKp strains: siderophores—yersiniabactin, aerobactin, and salmochelin; genotoxin—colibactin; and the hypermucoidy-associated locus (Lam et al., 2021). The presence of other virulence-associated genes such as *peg344*, *shiF*, and *luxR* was also considered in the selection process (Ye et al., 2016; Arena et al., 2022; Chen et al., 2023).

A total of 27% (*n* = 24) of the isolates originated from invasive samples/invasive devices (blood, punctate, cannula, drain, and broncho-alveolar lavage) and 73% (*n* = 65) from other clinical samples (urine, wound, and tracheal aspirate).

Of the 89 isolates, 32 were selected for long-read sequencing to enable comparative analysis of mobile genetic elements. Selection was based on phylogenetic analysis of short-read sequencing data.

For closely related isolates, we aimed to include samples from all hospitals represented within the corresponding phylogenetic cluster.

### 2.2 Phenotypic characterisation of the isolates

The isolates were identified using a MALDI Biotyper® sirius System (Bruker, Bremen, Germany). Disk diffusion synergism test was used for phenotypic screening of carbapenemase production according to the previously described in-house protocol (Buzgó et al., 2025). The disk diffusion susceptibility test was performed in accordance with the disk diffusion methodology recommended by EUCAST (European Committee on Antimicrobial Susceptibility Testing; EUCAST, 2023). The evaluation of synergism is based on assessing distortion of the inhibition zones to determine the synergistic effect of antibiotic discs (Buzgó et al., 2025).

For isolates with detected mgrB mutations, colistin susceptibility was assessed by broth microdilution method (Bruker UMIC®, Bremen, Germany) to confirm the predicted colistin-resistant phenotype. The test was conducted according to the manufacturer’s instructions, and the results were interpreted in accordance with EUCAST guidelines (EUCAST, 2024).

The hypermucoviscous phenotype was screened using string test. The isolates were cultured under standard conditions on 5% sheep blood Columbia agar (Biolab Zrt., Budapest, Hungary) and incubated at 37 °C overnight. Inoculation loop was used to stretch a mucous filament from a single colony, and the test was considered positive if the resulting filament was ≥5 mm in length (Russo et al., 2018; Arcari and Carattoli, 2023).

### 2.3 Whole-genome sequencing

#### 2.3.1 Short-read sequencing

Short-read sequencing was performed on Illumina platform (MiSeq and NextSeq devices) following the protocol described in the study (Buzgó et al., 2025). Genomic DNA libraries were prepared with DNA Prep Kit (Illumina, San Diego, CA, USA).

#### 2.3.2 Long-read sequencing

Genomic DNA was extracted using the Quick-DNA™ Fungal/Bacterial Kit (Zymo Research, USA), a spin column-based method. DNA concentration and purity were determined using a NanoDrop™ Lite spectrophotometer (Thermo Fisher Scientific, USA) and a Qubit™ Fluorometer V4. In addition, the quality of the extracted DNA was assessed using gel electrophoresis to detect extensive DNA fragmentation as well as RNA and protein contamination. No further purification or RNase treatment was performed. Two sequencing libraries were prepared using the Native Barcoding Kit SQK-NBD114.96 (Oxford Nanopore Technologies, UK) following the manufacturer’s instructions. As a negative control, UltraPure™ DNase/RNase-Free Distilled Water (Invitrogen, catalogue number 10977-035, USA) was used. According to the library preparation protocol, 400 ng of DNA from each sample was used to ensure an equimolar input. Sequencing was performed on a MinION Mk1C device (Oxford Nanopore Technologies, UK) using R10.4.1 flow cells (FLO-MIN114). The first library was sequenced once and yielded approximately 11.39 Gb of est. basepair based on fast-basecall. The second library was sequenced in multiple runs, resulting in a total output of approximately 9.6 Gb. The total amount of data from all sequencing runs was approximately 21 Gb.

### 2.4 Bioinformatics analysis

#### 2.4.1 Processing of short reads

The quality of the short-read dataset was first assessed using FastQC 0.11.9 (Andrews, 2010) and then filtered with fastp 0.20.1 (Chen et al., 2018) to remove low-quality reads and sequencing adapters and error-correct overlaps between read pairs. Reads were also trimmed to remove the first 10 and last five base pairs that had a relatively high error rate, as well as base pairs at either end of the reads that had a quality of less than 26. Sequencing errors were corrected using a k-mer frequency-based approach as implemented in Bloocoo 1.0.6 with default options (Drezen et al., 2014). The genome sequences were reconstructed then with SPAdes 3.13.1 (Prjibelski et al., 2020) using the—careful—cov-cutoff auto, and—only—assembler settings. The downstream analyses of the short-read assemblies, such as identification of plasmids, verification of species identification, screening for ARGs, VFs, and Incs, were performed as described above.

#### 2.4.2 Processing of long reads

**Figure 2:**
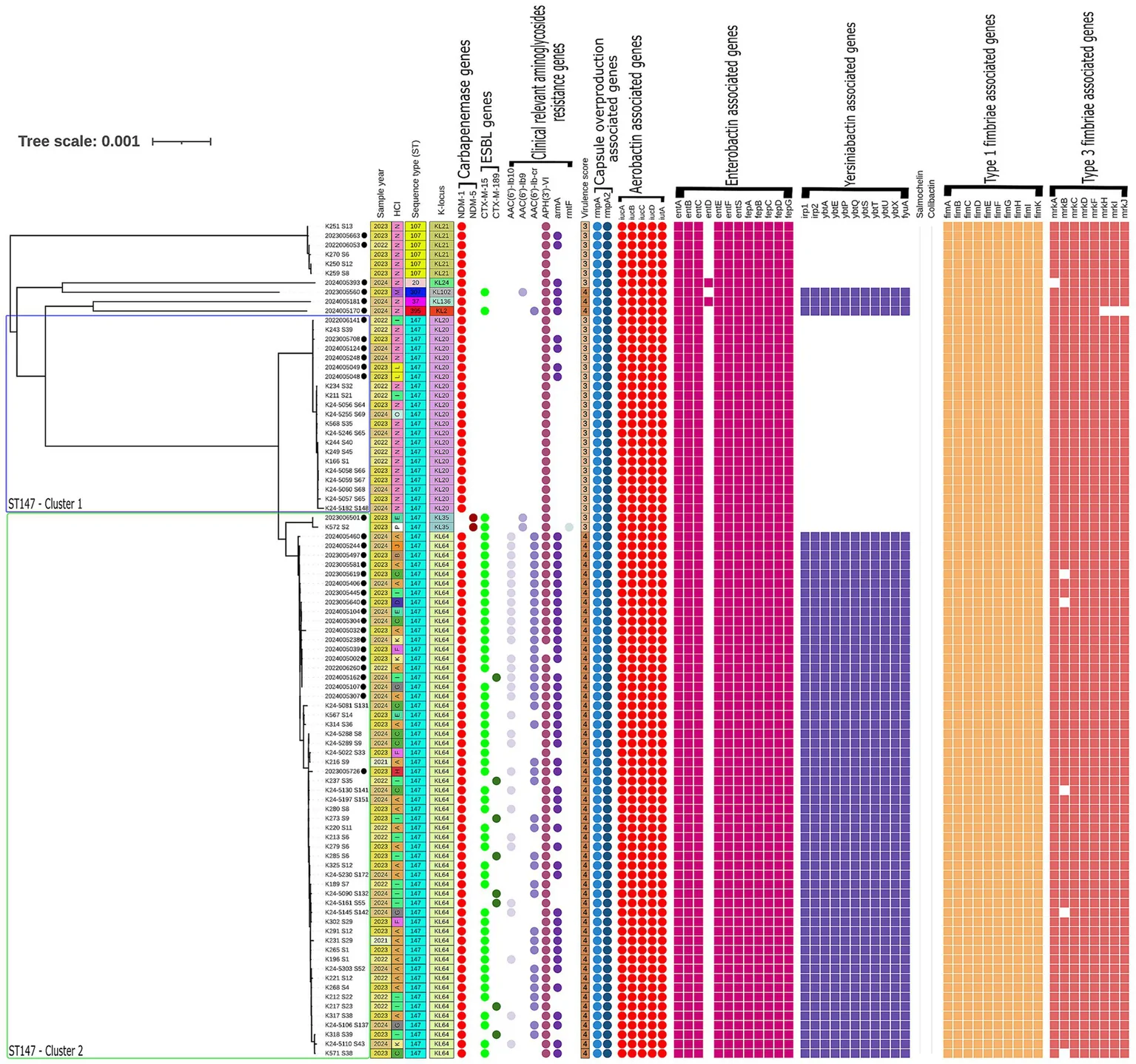
Phylogenetic tree of the hv(a)CpKp isolates included in our study (*n* = 89) and clinically relevant antibiotic resistance- and hypervirulence-associated genes carried by them. Isolates marked with black circle have been selected to long-read sequencing.

**Figure 3:**
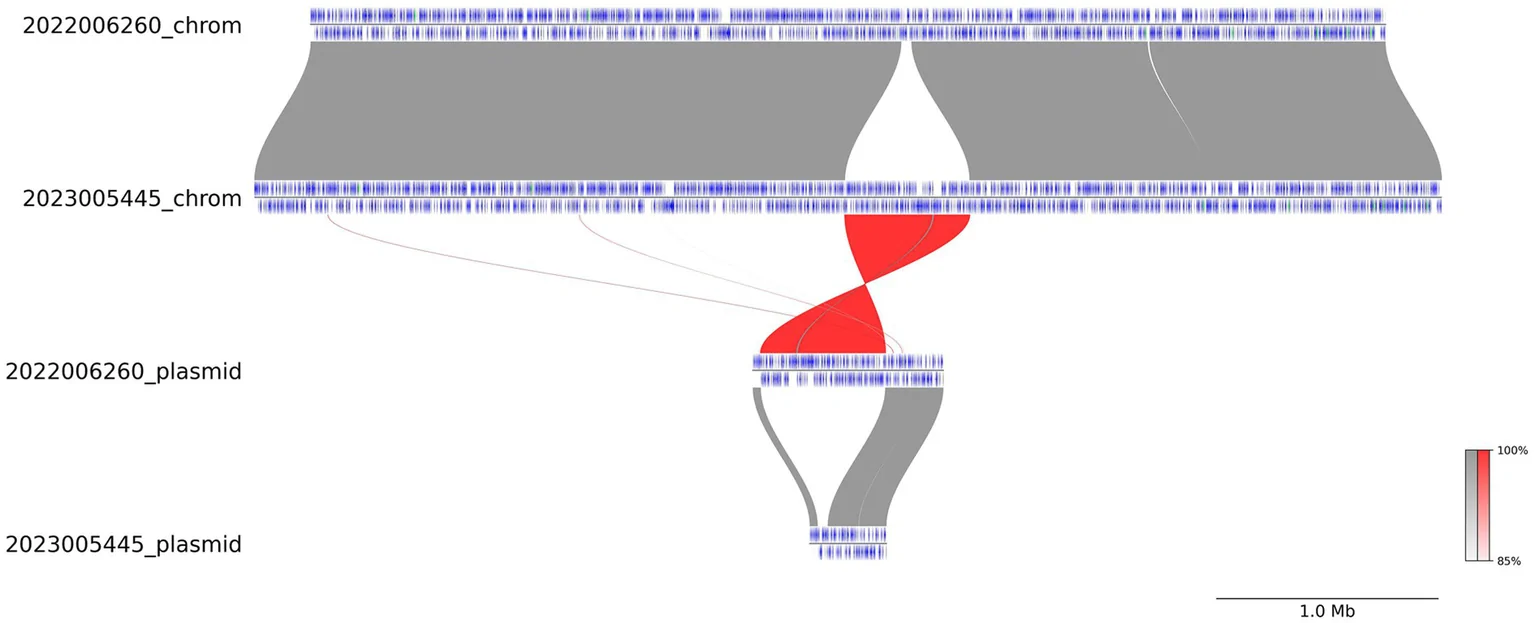
Site-specific recombination between the chromosome and the IncHI1B/IncFIB(Mar) megaplasmid in *K. pneumoniae* 2022006260 isolate.

**Figure 4:**
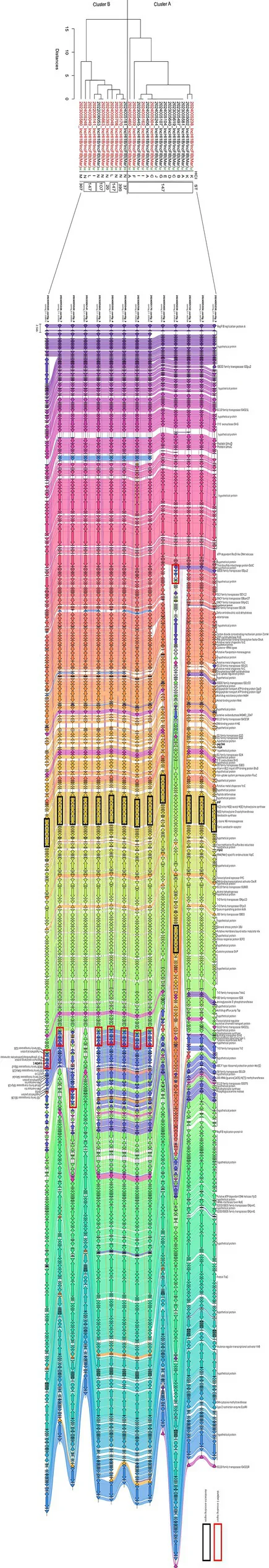
Dendrogram illustrates the pairwise DCJ distances of IncHI1B/IncFIB(Mar) plasmids (grouped by containment distance of ≤0.5) based on pling results. The structures of the representative isolates highlighted in red were visualised using clinker; V indicates plasmids carrying only virulence genes, while H denotes hybrid plasmids harbouring both virulence and *bla*NDM-1 genes.

Raw reads were basecalled with dorado 0.8.2 (Oxford Nanopore Technologies, Oxford, UK) using the super high accuracy model (dna_r10.4.1_e8.2_400bps_sup@v5.0.0). Reads were then filtered with Chopper 0.8.0 (De Coster and Rademakers, 2023) to remove sequencing adaptors and low-quality reads (q < 8). The quality of reads was controlled with NanoPlot 1.38.1 (De Coster and Rademakers, 2023). The genome sequences were assembled with Flye 2.9-b1768 (Kolmogorov et al., 2019) using the --nano-hq option and then polished with Racon 1.4.10(Vaser et al., 2017) and medaka 1.7.2 (Oxford Nanopore Technologies Ltd., 2018) using the model r104_e81_sup_g610. Polished assemblies were reoriented with dnaapler 1.0.1 (Bouras et al., 2024) to use the same start site of the circular contigs in downstream analyses. The quality of the assemblies was assessed with QUAST 5.0.2 (Gurevich et al., 2013) and BUSCO 5.2.2 (Simão et al., 2015) to ensure high contiguity and completeness, and possible contamination was checked with CheckM 1.2.2 (Parks et al., 2015). Contigs were also classified with kraken 2.1.2 (Wood et al., 2019) to verify species identification using the k2_pluspf database version 1/12/2024 (available at: https://benlangmead.github.io/aws-indexes/k2). Sequence types (STs) were identified using MLST 2.23.0 (Seemann, 2026a) with automatic scheme selection enabled to automatically find the optimal PubMLST (Jolley and Maiden, 2010) scheme. Plasmids were identified with Platon 1.7 (Schwengers et al., 2020). Plasmid mobility was predicted using MOB-typer v3.0.3 (Robertson and Nash, 2018), and our in-house tool, screen_tradb.py0.2.8 (Freytag et al., 2024), was used to identify plasmid transfer genes. Antimicrobial resistance genes (ARGs), virulence factors (VFs), and plasmid incompatibility groups (Incs) were identified using ABRicate 1.0.1 (Seemann, 2026b) CARD (Jia et al., 2017), VFDB (Chen et al., 2016), and PlasmidFinder (Carattoli et al., 2014) databases obtained, 2025-May-21. HvKp-specific VFs such as *peg344*, *luxR*, and *shiF* were screened using a custom database consisting of reference sequences identified in *K. pneumoniae* accessions CP182017.1; KC354802.1; AY378100.1; CP190187.1. Integrative mobile elements were identified with MeFinder 1.1.2 (Durrant et al., 2020). Genes were annotated with prokka 1.14.6 (Seemann, 2014) with default options, and the pangenome of the isolates was reconstructed with Panaroo 1.1.2 (Tonkin-Hill et al., 2020) using --mode moderate. The core genome alignment was used for the phylogenetic reconstruction of the isolates with IQtree 2.1.4-beta (Nguyen et al., 2015) with automatic nucleotide substitution model selection and using 1,000 aLRT and 1,000 rapid bootstrap replicates to assess the robustness of the tree topology. Single nucleotide polymorphism (SNP)-based analysis was performed on a representative subset of isolates, all polymorphisms from the core genome were extracted using snp-sites v2.5.1 (Page et al., 2016) and a minimum spanning network was reconstructed for the representative isolates, with all gap sites excluded to reduce ambiguity. Capsule and O antigen type was predicted using Kaptive 3.0.0b1 (Wyres et al., 2016; Lam et al., 2022; Stanton et al., 2025) as implemented in Kleborate 3.1.3 (Lam et al., 2021), which was also used to confirm the presence of *ICEKp* virulence loci, virulence plasmid-associated loci, and antimicrobial resistance determinants (acquired genes, SNPs, gene truncations, and intrinsic *β*-lactamases). Comparative analysis of the plasmids was performed using clinker 0.0.31, and a subset of the Figures 2–4 presented in this study were generated from these clinker-derived comparisons (Gilchrist and Chooi, 2021) to assess the gene content of the different plasmids and pling 2.0.0 (Frolova et al., 2024) to assess the evolutionary distances between the different plasmids.

Comparative analyses were performed by grouping the plasmids based on the containment distance value by pling 2.0.0. The plasmids are in one group whose containment distance (CD) was <0.5. The CD, or content/overlap distance, indicates the difference between the sequences of two plasmids—that is, the proportion of the smaller plasmid’s sequence that is not present in the larger plasmid. The dendrograms in Figures 2–4 are based on the double cut and join (DCJ) distances calculated by pling.

The DCJ metric expresses the number of structural changes, such as rearrangements and insertions or deletions, between the sequences of two compared plasmids. If this value is < 5 and the CD value is also < 0.5, the plasmids are considered to belong to the same subcommunity. Non-circular contigs and contigs with triple-replicon (IncFIB(Mar)_1_pNDM-Mar / IncHI1B_1_pNDM-MAR / IncFIB(pQil)_1_pQil; and IncFIB(Mar)_1_pNDM-Mar / IncR_1 / IncHI1B_1_pNDM-MAR), which pling was unable to distinguish precisely, were excluded from the analysis.

In the case of the detected >800 kbp IncHI1B/IncFIB(Mar) plasmid, we evaluated chromosomal and plasmid read depth and alignment support in detail. Chromosomal read depth was visualised using dep v0.1.0 (gpsz/dep: Draw depth plot of bam, 2024). Recombination between the chromosome and the plasmid was assessed using pyGenomeViz v0.4.4 with the pgv-mummer workflow to visualise genomic synteny and structural rearrangements (Shimoyama, 2024).

**Figure 5:**
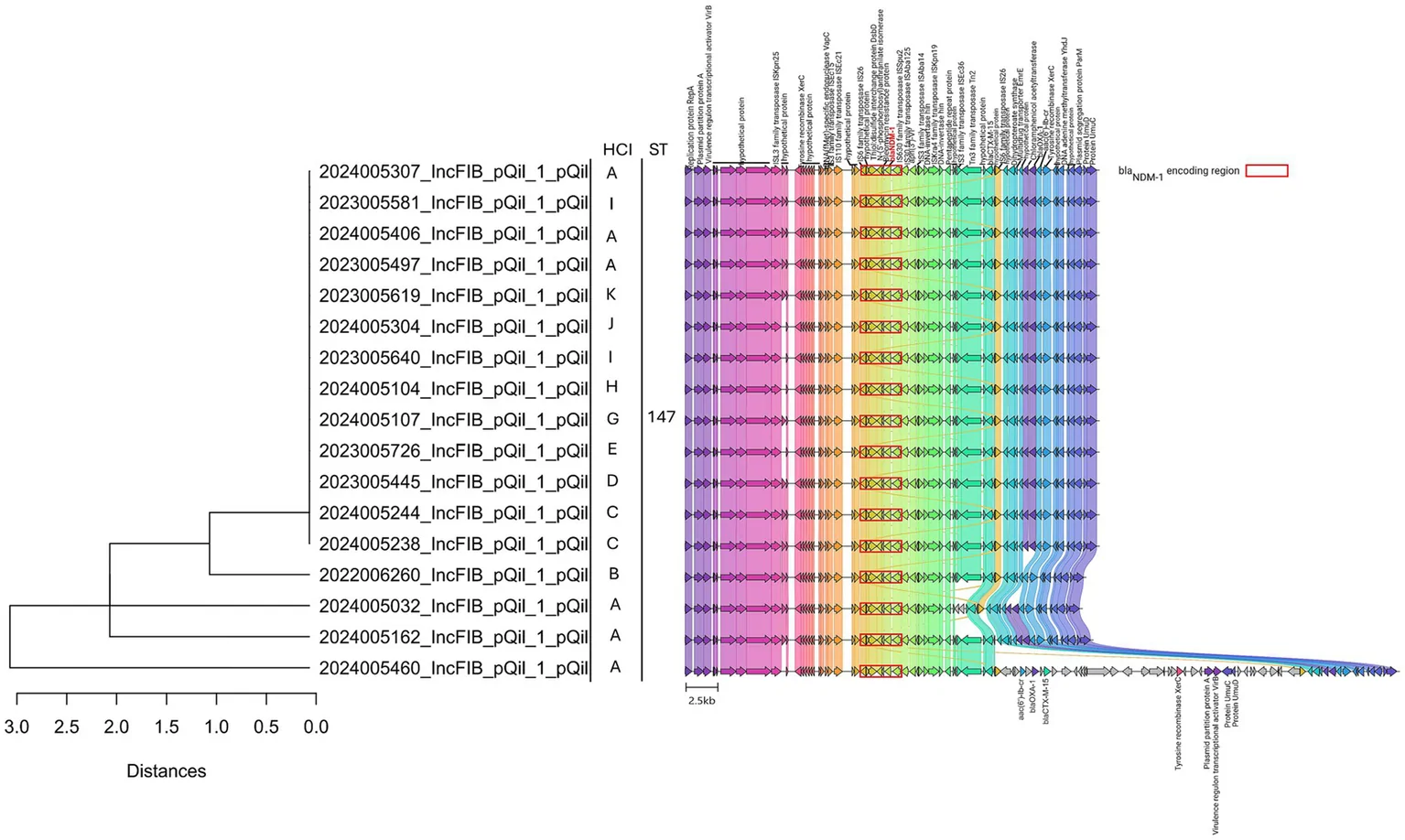
Dendrogram illustrates the pairwise DCJ distances of IncFIB(pQil) plasmids (grouped by containment distance of ≤ 0.5) based on Pling results.

The figures were edited with Inkscape 1.4.2 (available at https://inkscape.org/) to increase readability if necessary. Figure 5 was visualised with Interactive Tree of Life (iTOL v6; Letunic and Bork, 2024).

## 3 Results

### 3.1 Characterisation of isolates

#### 3.1.1 Population structure

The majority of isolates (79/89) belonged to sequence type (ST) 147. Six isolates belonged to ST107, and the remaining singletons belonged to ST20, ST37, ST307, and ST395. Within ST147, we were able to distinguish two clusters. Cluster 1 comprised 21/79 isolates that mostly not carried yersiniabactin-associated genes, and 16/21 of them originated from a HCI from the eastern part of the country. Cluster 2 comprised the majority of isolates, 58/79, all of which carried yersiniabactin-associated genes and originated from 13 HCIs surrounding the capital city (Figure 2; detailed genetic characteristics of the isolates are provided in Supplementary Table 1).

SNP analysis identified more than 24,000 SNPs between different sequence types (STs). Within ST147, two distinct lineages were separated by 2,768 SNPs, consistent with the phylogenetic tree. Pairwise SNP distances within the major groups ranged from 11 to 2,521, in concordance with the phylogenetic tree, which shows a similar structure based on both short- and long-read sequencing data (The results of the SNP analysis are presented in Supplementary Figure 1).

Pangenome variability analysis identified 7,776 genes, of which 4,412 (56.7%) constituted the core genome (present in ≥99% of isolates), while most of the remaining genes belonged to the cloud genome (*n* = 2,109), present in less than 15% of isolates. Clustering based on the presence or absence of genes correlated with the observed sequence types and the described phylogenetic structure, which is based on core genome alignment. Gene accumulation analysis, based on five independent random resampling at each sampling depth, reached a clear plateau, indicating that the inclusion of additional isolates would contribute few novel genes to the pangenome (Supplementary Figures 2, 3).

#### 3.1.2 Capsular type

The isolates belonging to ST147 Cluster 1 (n = 21) carried the KL20 capsular locus (associated with *wzi-95 allele*) in combination with the O2v1 O antigen locus and were originated from four HCIs. The isolates assigned to ST147 Cluster 2 (n = 56) harboured the KL64 capsular locus (*wzi-64 allele*) together with O2v1 and originated from 11 HCIs, while the two remain ST147 isolates carried the KL35 locus (*wzi-23 allele*) in combination with the O5 locus and originated from two HCIs.

All six ST107 isolates carried the KL21 capsular locus (*wzi-21 allele*) associated with O2v1 and originated from a single HCI. The single ST20 isolate carried the KL24 locus (*wzi-24 allele*) in combination with O2v1. The ST37 isolate harboured the KL136 locus (*wzi-123 allele*) together with O2v2, the ST307 isolate carried the KL102 locus (wzi-173 allele) with O2v2, and the ST395 isolate possessed the KL2 locus (*wzi-2 allele*) in association with O2v1 (Figure 2; Supplementary Table 1).

#### 3.1.3 Antibiotic resistance determinants

The *bla*_NDM-1_ carbapenemase gene was detected in most isolates (87/89), whereas the *bla*_NDM-5_ gene was found in two isolates. The following ESBL genes conferring resistance to cephalosporins were identified: *bla*_CTX-M-15_ (*n* = 52) and *bla*_CTX-M-189_ (*n* = 8). Other *β*-lactamases of lower clinical relevance were also identified: *bla*_OXA-1_ (*n* = 56), *bla*_OXA-9_ (*n* = 54), *bla*_TEM-150_ (*n* = 54), and *bla*_TEM-1_ (*n* = 3). Therapeutically relevant aminoglycoside resistance-associated genes were also identified: *aph(3′)-VI* (*n* = 88), *aac(6′)-Ib-cr* (*n* = 40), *aac(6′)-Ib10* (*n* = 30), *aac(6′)-Ib9* (*n* = 3), *armA* (*n* = 53), and *rmtF* (*n* = 1; Figure 2).

Mutations in the *mgrB* gene were detected in 11 isolates. Among these isolates, 10 were resistant to colistin (MIC = > 8 μg/mL), while one isolate was susceptible to colistin (MIC = 0.25 μg/mL).

The following fluoroquinolone resistance genes were identified: *qnrS1* (*n* = 83), *qnrB17* (*n* = 2), and *qnrB1* (*n* = 1). Fluoroquinolone resistance-associated mutations were also detected, mostly co-occurring with the listed fluoroquinolone resistance genes: *GyrA-83 L* and *ParC-80C* (*n* = 81; DNA gyrase and topoisomerase IV mutations). All identified resistance genes are listed in Supplementary Table 1.

#### 3.1.4 Virulence factors

The presence of the *rmpA*, *rmpA2*, *peg344*, *shiF*, *luxR,* and aerobactin-related genes (*iucA-D and iutA*) was detected in all isolates. The other detected hypervirulence-associated genes were integrated into the chromosomes. Yersiniabactin-related genes (*irp1-2*, *ybtAEPQSTUX*, and *fyuA*) were detected in 59 isolates. We identified three yersiniabactin lineages: ybt9 (*n* = 57; 56 belonging to ST147 and one to ST37), ybt10 (*n* = 1; ST307), and ybt16 (*n* = 1; ST395). The chromosomal integration of ybt9 may have been facilitated by the integrative conjugative element *ICEKp3*, ybt10 by *ICEKp4*, and ybt16 by *ICEKp12*. The enterobactin-related genes, the *entA-C*, *entE*, *entS*, *fepA-D,* and *fepG* were present in all isolates, while the *entD* gene was detected in only two isolates. Salmochelin and colibactin-related genes were not detected in the isolates. According to the Kleborate pipeline, 30/89 isolates had a virulence score of 3, while 59/89 isolates had a virulence score of 4. Type 1 fimbriae-related genes (*fimA–K*) were detected in all isolates. Type 3 fimbriae-related genes (*mrkA–J*) were also detected in all isolates, except that *mrkA* was absent in one isolate, *mrkB* in five isolates, and *mrkH–J* in one isolate (Figure 2; Supplementary Table 1). Based on the results of the string test, 22.5% (*n* = 20) of the isolates exhibited a hypermucoviscous phenotype (Supplementary Table 1).

### 3.2 Plasmid analysis based on long-read sequencing

Based on the long-read sequencing results of 32 strains, a total of 88 plasmids were identified. The most frequent incompatibility groups (Inc) were IncHI1B_1_pNDM-MAR/IncFIB(Mar)_1_pNDM-Mar (*n* = 29; hereafter IncHI1B/IncFIB(Mar)); IncR_1 (*n* = 21; hereafter IncR); and IncFIB(pQil)_1_pQil (*n* = 18; hereafter IncFIB(pQil)). These three dominant plasmid families carried most clinically relevant antimicrobial resistance genes. Detailed structural analysis of these plasmids revealed the mobilisation patterns of resistance and virulence genes. The other identified plasmids, along with detailed information on their size, coverage, circularity, and contig assignment, are provided in Supplementary Table 2.

### 3.3 Integration of chromosomal regions into a plasmid

An IncHI1B/IncFIB(Mar) plasmid of over 800 kbp was detected in isolate 2022006260. Comparison with a closely related strain (2023005445) revealed that a specific genomic region present in 2023005445 was missing from the chromosome of 2022006260 but had been integrated into its plasmid. In contrast, this region is absent from the plasmid of strain 2023005445. This demonstrates the integration of the chromosomal region into the plasmid (Figure 3; see Supplementary Figure 4 for the plasmid structure). The analysis of read depth and alignment support showed that the plasmid exhibits slightly higher read depth than the chromosome (approximately 102 × compared to the chromosome’s 72×), with a uniform coverage pattern across the contig (Supplementary Figures 5, 6). In addition, read alignments provided strong support for the inferred structure, with high-confidence mappings observed at the putative recombination junctions on both the chromosome and the plasmid.

### 3.4 Comparative analysis of prevalent plasmid types

#### 3.4.1 IncHI1B/IncFIB(mar) plasmids—carbapenemase—and hypervirulence-associated genes

The *rmpA*, *rmpA2*, *peg344*, *shiF*, *iucA-D,* and *iutA* hva genes were predominantly carried on the IncHI1B/IncFIB(Mar), which were detected in 29 out of 32 isolates. In two other cases, other multiple replicons linked to these plasmids also carried these genes (IncHI1B/IncFIB(Mar)/IncFIB(pQil); IncHI1B/IncFIB(Mar)/IncR).

We identified plasmids simultaneously carrying hva and carbapenemase genes—designated as hybrid plasmids—in 13 of 32 isolates. Most of these (11 out of 12) carried the following resistance genes in addition to hva genes: *bla*_NDM-1_, *aph(3′)-VI*, and *armA*. The remaining plasmid harboured genes *bla*_NDM-1_, *bla*_CTX-M-15_, *aac(6′)-Ib-cr*, and *armA*.

Almost all of these IncHI1B/IncFIB(Mar) plasmids were predicted to be conjugative and carried complete set of transfer genes (*TraA, TraC, TraD, TraE, TraF, TraG, TraH, TraI, TraJ, TraK, TraN, TraU, TraV, TraW*, and *TrbN*), except for isolate 2,024,005,460, which lacked *TraA, TraE,* and *TraK.* The >800 kb IncHI1B/IncFIB(Mar) plasmid of isolate 2022006260 was not predicted to be conjugative; however, it still harboured a partial set of transfer genes (*TraA, TraC, TraD, TraE, TraG, TraK, TraN, TraQ, TraU, TraV*, and *TraW*; Supplementary Table 2).

Notably, we identified a hybrid plasmid in one additional case of IncFIB(K), which, in addition to hva genes, harboured *bla*_NDM-5_, *bla*_CTX-M-15_, and *aph(3′)-VI* resistance genes (Table 1).

**Table 1:** Distribution of clinical relevant antimicrobial resistance- and hypervirulence-associated genes on different plasmid types based on long-read sequencing of 32 isolates.

HCI	SAMPLE ID	ST	Plasmid encoded relevant β-lactamase genes	Plasmid encoded clinical relevant aminoglycoside resistance-associated genes	Plasmid encoded hypervirulence-associated genes
A	2023005581	147	*bla*_NDM-1_ *bla*_CTX-M-15_	*aph(3′)-VI aac(6′)-Ib-cr*	*rmpA*, *rmpA2*, *iucA-D*, *iutA*, *peg344*, *shiF*
	2024005307	147			
	2024005406	147			
C	2023005619	147			
	2024005304	147		*aac(6′)-Ib10*	
D	2023005640	147			
G	2024005107	147			
H	2023005726	147			
I	2023005445	147		*armA*	
J	2024005244	147			
K	2024005002	147			
	2024005238	147			
A	2022006260	147	*bla*_NDM-1_ *bla*_CTX-M-15_	*armA*	*rmpA*, *rmpA2*, *iucA-D*, *iutA*, *peg344*, *shiF*
				*aac(6′)-Ib-cr aac(6′)-Ib10*	
	2024005032	147	*bla*_NDM-1_ *bla*_CTX-M-15_	*aph(3′)-VI*	*rmpA*, *rmpA2*, *iucA-D*, *iutA*, *peg344*, *shiF*
				*aac(6′)-Ib-cr*	
				*aac(6′)-Ib-cr*	
				*aac(6′)-Ib10*	
				*armA*	
	2024005460	147	*bla*_NDM-1_ *bla*_CTX-M-15_	*aph(3′)-VI*	*rmpA*, *rmpA2*, *iucA-D*, *iutA*, *peg344*, *shiF*
				*aac(6′)-Ib10*	
				*aac(6′)-Ib-cr*	
			*bla*_CTX-M-15_	*armA*	
B	2023005497	147	*bla*_NDM-1_ *bla*_CTX-M-15_	*aph(3′)-VI*	*rmpA*, *rmpA2*, *iucA-D*, *iutA*, *peg344*, *shiF*
				*aac(6′)-Ib-cr*	
				*armA*	
				*aac(6′)-Ib10*	
E	2023006501	147	*bla*_NDM-5_ *bla*_CTX-M-15_	*aph(3′)-VI*	*rmpA*, *rmpA2*, *iucA-D*, *iutA*, *peg344*, *shiF*
				*aac(6′)-Ib9*	
	2024005104	147	*bla*_NDM-1_ *bla*_CTX-M-15_	*aph(3′)-VI*	*rmpA*, *rmpA2*, *iucA-D*, *iutA*, *peg344*, *shiF*
				*aac(6′)-Ib-cr*	
				*aac(6′)-Ib10*	
				*armA*	
F	2024005039	147	*bla*_NDM-1_ *bla*_CTX-M-15_	*aac(6′)-Ib-cr armA*	*rmpA*, *rmpA2*, *iucA-D*, *iutA*, *peg344*, *shiF*
	2022006141	147	*bla*_NDM-1_	*aph(3′)-VI*	*rmpA*, *rmpA2*, *iucA-D*, *iutA*, *peg344*, *shiF*
	2024005162	147	*bla*_NDM-1_ *bla*_CTX-M-189_	*aph(3′)-VI*	*rmpA*, *rmpA2*, *iucA-D*, *iutA*, *peg344*, *shiF*
				*aac(6′)-Ib-cr*	
				*aac(6′)-Ib10*	
				*armA*	
L	2024005048	147	*bla*_NDM-1_	*aph(3′)-VI armA*	*rmpA*, *rmpA2*, *iucA-D*, *iutA*, *peg344*, *shiF*
	2024005049	147			
N	2022006053	107			
	2023005663	107			
	2023005708	147			
	2024005124	147			
	2024005181	37			
	2024005393	20			
	2024005170	395	*bla*_NDM-1_	*aph(3′)-VI armA*	*rmpA*, *rmpA2*, *iucA-D*, *iutA*, *peg344*, *shiF*
			*bla*_CTX-M-15_	*aac(6′)-Ib-cr*	
	2024005248	147	*bla*_NDM-1_	*aph(3′)-VI armA*	*rmpA*, *rmpA2*, *iucA-D*, *iutA*, *peg344*, *shiF*
			*bla*_NDM-1_	*aph(3′)-VI*	
M	2023005560	307	*bla*_NDM-1_	*aph(3′)-VI armA*	*rmpA*, *rmpA2*, *iucA-D*, *iutA*, *peg344*, *shiF*
			*bla*_CTX-M-15_	*aac(6′)-Ib9*	
Colour	Plasmid type				
	IncHI1B/IncFIB(Mar)				
	IncHI1B/IncFIB(Mar)/IncR				
	IncHI1B/ IncFIB(Mar)/IncFIB(pQil)				
	IncFIB(K)				
	IncFIB(pQil)				
	IncR				
	Not detected on the same contig of the listed antibiotic resistance gene of the Inc. plasmid group.				

The first four digits indicate the sample year of isolates.

In the comparative analysis of 26 IncHI1B/IncFIB(Mar) plasmids, the dendrogram revealed two major clusters at the first principal branching point, defined by DCJ distances >5: Cluster A and Cluster B. Within each cluster, DCJ values were <5. Cluster A comprises 15 plasmids, all but one carrying only hva genes. Cluster B comprised 11 plasmids, and with one exception, all were hybrid plasmids. In summary, mainly plasmids carrying only virulence genes were separated from hybrid plasmids. Hybrid plasmids showed greater diversity than plasmids carrying only virulence genes (Figure 4).

The genetic environment of the aerobactin and *bla*_NDM-1_ genes was investigated in detail in these 26 isolates. The aerobactin-encoding segment of hybrid plasmids was located between *IS1N* (*IS1*) and *ISNpu13* (*Tn3 family*) transposons and contained genes involved in aerobactin biosynthesis (e.g., aerobactin synthase and ferric aerobactin receptor), iron and B12 transport systems, transcriptional regulation (transcriptional repressor PifC, DNA-binding transcriptional activator DecR), and metabolic and stress response (alcohol dehydrogenase, tRNA(fMet)-specific endonuclease VapC, and free methionine R-sulphoxide reductase). The *bla*_NDM-1_ encoding segment was located downstream of the aerobactin-encoding segment. The region was part of a composite transposon bounded by *IS26* (*IS6 family*)–*ISSpu2* (*IS630 family*) and carried bleomycin resistance protein and N-acetyltransferase genes upstream of *bla*_NDM-1_. Downstream of this segment, the *aph(3′)-VI* gene appeared between the insertion elements *ISAba125* (*IS30 family*) and *ISAba14* (*IS3 family*). The *bla*_NDM-5_ and aerobactin-encoding gene region detected on the IncF(K) plasmid was genetically identical to *bla*_NDM-1_ and aerobactin-encoding gene region found on IncHI1B/IncFIB(Mar). The *bla*_NDM-1_ gene encoding region was identical across all plasmid types, indicating active, insertion element-mediated horizontal dissemination. The aerobactin coding region in plasmids carrying only hva genes was identical to the structure of the same region in hybrid plasmids (Figure 4).

#### 3.4.2 IncFIB(pQil) plasmids—stable multidrug resistance modules

In the case of the 17/32 isolates, the *bla*_NDM-1_ was located on the IncFIB(pQil) together with *bla*_CTX-M-15_, *aph(3′)-VI, and aac(6′)-Ib-cr*, whereas hva genes carried separately on IncHI1B/IncFIB(Mar) plasmids.

The analysis of 17 IncFIB(pQil) plasmids revealed that DCJ distances within the group were low ranging from 0 to 3. Most of the plasmids (13 out of 17) had a DCJ distance of 0 from each other, indicating that these plasmids were structurally identical. One plasmid differed by a DCJ distance of 1, two plasmids by 2, and one plasmid by 3, the latter containing a structurally unique insertion.

The *bla*_NDM-1_ gene encoding region was highly conserved across all types of plasmids, indicative of active horizontal dissemination. The *bla*_CTX-M-15_-encoding segment was located downstream of these regions and was bounded by *Tn3* (*Tn2 family*) and *IS26* (*IS6 family*) transposases. The downstream region of *bla*_CTX-M-15_ segment includes *aac(6′)-Ib-cr, cat*, *EmrE* multidrug efflux pump, *bla_OXA-1_* genes. In one isolate, duplication of *aac(6′)-Ib-cr* was also observed.

Most IncFIB(pQil) plasmids were highly similar, exhibited conserved gene structures, and were not predicted to be conjugative, which suggests that the same plasmid spread with the same clones (Table 1; Figure 5; Supplementary Table 2).

#### 3.4.3 IncR plasmids—variable antibiotic resistance genes

The IncR plasmid type was detected in 21 out of 32 isolates and harboured variable aminoglycoside and other antibiotic resistance genes.

Most IncR plasmids (15/21) carried *aac(6′)-Ib-cr*, streptomycin 3″-adenylyltransferase (*aad*A), *bla_OXA-18_*, and *bla*_TEM_-type *β*-lactamase genes within a single segment. This segment was located between *IS26* (*IS6 family*) and *ISPa38* (*Tn3 family*) transposases. Two IncR plasmids carried the *bla*_CTX-M-15_ gene. In isolate 2,024,005,170, the *bla*_CTX-M-15_ gene was located between *IS26* (*IS6*) and *Tn2* (*Tn3*) transposases, whereas in isolate 2,023,006,501, it was between the *Tn2* (*Tn3*) transposase and the *ISEcp1* (*IS1380*) insertion element. The IncR plasmid of 2,022,006,141 carried a structurally identical *bla*_NDM-1_ and *aph(3′)-VI* segment to those found in IncFIB(pQil) and IncHI1B/IncFIB(Mar), and three plasmids did not carry any antibiotic resistance gene.

**Figure 6:**
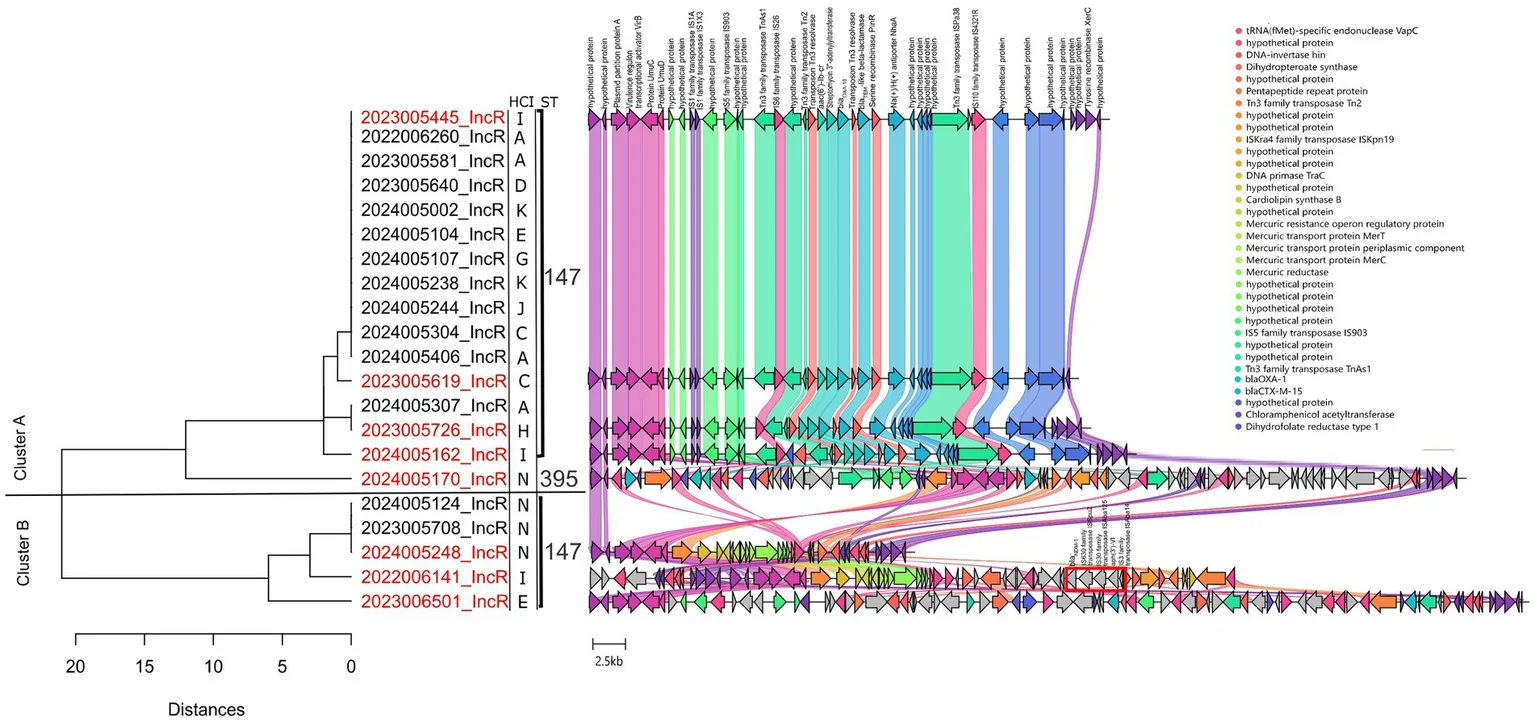
Dendrogram illustrates the pairwise DCJ distances of IncR plasmids (grouped by containment distance of ≤ 0.5) based on pling results, and the structure of the representative isolates highlighted in red is visualised using clinker.

From the comparative analysis of these 21 plasmids, we identified two clusters—Cluster A and Cluster B—at the first major branching point of the dendrogram, based on DCJ distances greater than 5. Cluster A contained 16 plasmids, 11 of which had a DCJ distance of 0, indicating they were identical. Cluster B included five plasmids, three of which were structurally identical, also with a DCJ distance of 0 (Table 1; Figure 6). The two clusters also differed in conjugative potential. All Cluster A plasmids were predicted to be conjugative, whereas only one plasmid from the 2023006501 isolate in Cluster B was predicted to be conjugative; the remaining Cluster B plasmids were non-mobilisable (Supplementary Table 2).

## 4 Discussion

Our study is, to our knowledge, the first comprehensive review of the genetic characterisation of hv(a)CpKp strains in Hungary. The emergence of hv(a)CpKp strains represents a new public health threat in Hungary. This concern is further reinforced by the fact that most strains included in the study (79/89) belonged to the high-risk ST147 clone. To place our findings in a broader epidemiological context, it is important to consider the global evolution and spread of the ST147 clone.

ST147 is a globally distributed high-risk clone, endemic in India, Greece, Italy, and North African countries. It has caused healthcare-associated infections in many regions, including Europe, the Far East, Australia, and South America. In the early 2000s, the *bla*_CTX-M-15_ gene had already been acquired on IncF or IncR plasmids and marked the beginning of the clone’s global expansion (Peirano et al., 2020). Around this period, these strains also appeared in Hungary and were associated with hospital outbreaks (Damjanova et al., 2008). Subsequently, the acquisition of various carbapenemase genes in the mid to late 2000s led to the emergence of distinct carbapenemase-associated clades, including *bla*_VIM_- and *bla*_KPC_-producing ST147 strains in Greece, *bla*_OXA-48_-producing strains in North Africa, and *bla*_NDM_- and *bla*_OXA-181_-producing strains in India (Peirano et al., 2020). In 2018–2019, *bla*_NDM-1_-producing ST147 caused a large outbreak in Tuscany, Italy, with a total of 1,495 cases (Tavoschi et al., 2020). In our previous nationwide study (2021–2023), the *bla*_NDM-1_-producing ST147 CpKp strains were predominant in Hungary (Buzgó et al., 2025). Furthermore, between 2020 and 2021, the hypervirulent, multidrug-resistant ST147 strains had already caused an outbreak at the tertiary care University Hospital of Pisa in Tuscany, Italy (Falcone et al., 2022). Our findings suggest a similarly rapid evolutionary trajectory among ST147 strains and provide evidence for the interregional dissemination of ST147 hv(a)CpKp strains in Hungary. The coexistence of detected carbapenemase and hva genes is particularly concerning as it may contribute to antimicrobial resistance and is potentially associated with increased virulence. These findings indicate the long-term persistence and considerable public health significance of the ST147 clone in Hungary.

Among the ST147 isolates, we identified two distinct clusters with clear regional distribution. The separation of the two clusters was also supported by the SNP analysis, indicating that two ST147 lineages are circulating in Hungary. Furthermore, plasmid analysis of representative isolates revealed distinct patterns of hva gene acquisition characteristic of the two clusters. In Cluster 1 (eastern region), both hva and carbapenemase genes were acquired via conjugative hybrid IncHI1B/IncFIB(Mar) plasmids. In Cluster 2 (capital region), the carbapenemase gene was located on a non-mobilisable IncFIB(pQil) plasmid, suggesting vertical transmission of strains. However, these isolates also acquired hva genes through conjugative IncHI1B/IncFIB(Mar) plasmids. Overall, conjugative IncHI1B/IncFIB(Mar) plasmids played a key role in the horizontal transmission of hva genes. From an evolutionary perspective, the convergence of classic and hypervirulent pathotype processes may occur through three main mechanisms: acquisition of multidrug resistance determinants by hvKp strains; acquisition of hypervirulence plasmids by MDR-Kp strains; and acquisition of hybrid plasmids carrying both resistance and virulence genes (Arcari and Carattoli, 2023). Our results demonstrate the latter two scenarios: MDR-Kp strains in the capital region of Hungary acquired hypervirulence plasmids, while strains from the eastern region acquired hybrid plasmids.

In recent years, an increasing number of countries have reported the presence of IncHI1B/IncFIB(Mar) hybrid plasmids. International studies similarly report IncHI1B/IncFIB(Mar) plasmids as major carriers of hypervirulence genes. The origin of these hybrid plasmids can be traced back to the pNDM-Mar plasmid, carrying the same IncHI1/IncFIB replicons first identified in 2011 in a ST15 *K. pneumoniae* isolate from Morocco. The hva gene repertoire of these plasmids is likely derived from archetypal, best characterised, highly conserved pK2044- and pLVpK-like hvKp virulence plasmids (Tang et al., 2020; Arcari and Carattoli, 2023). A large-scale genomic analysis of *K. pneumoniae* genomes (*n* = 2,578) indicates that over 90% of European and Asian IncFIB(Mar) plasmids carry hva genes (Sun et al., 2025), while IncHI1B plasmids represent one of the most common hv-associated plasmid backbones (Spadar et al., 2023). Hybrid resistance–virulence plasmids have been described in multiple countries. Shapovalova et al. (2024) identified in 38 *K. pneumoniae* (21 Russian HCIs) carbapenemase genes (*n* = 21 *bla*_NDM-1_, *n* = 9 *bla*_OXA-48-like_, and *n* = 1 *bla*_NDM_) and virulence genes (*iucA-D*, *iutA*, *rmpA*, *rmpA2*, and *peg344*) on IncHI1B/IncFIB(Mar) plasmids (Shapovalova et al., 2024). Similar results were obtained in Italy, where the IncHI1B/IncFIB(Mar) hybrid plasmids were identified in 36 ST147 multidrug-resistant hvKP isolates (Falcone et al., 2022). In the United Kingdom, IncHI1B/IncFIB(Mar) hybrid plasmids were first detected in ST147 isolates in 2016, with significant spread reported between 2022 and 2023 (Turton et al., 2024). All of these further underscore the epidemiological significance of the Hungarian ST147 strains, and the emergence of hv(a)CpKp strains appears to reflect a broader European trend.

Plasmid comparison analysis showed that only hva genes carrying IncHI1B/IncFIB(Mar) plasmids formed a distinct group separate from hybrid plasmids, which exhibited greater diversity. Isolates carrying only hva genes harbouring IncHI1B/IncFIB(Mar) plasmids belonged to ST147 Cluster 2 and were highly related, consistent with clonal dissemination.

The diversity of hybrid plasmids may be attributed to their presence across multiple sequence types, including ST147, ST107, ST395, ST20, and ST37. At the same time, the hybrid plasmid carrying ST147, which belongs to Cluster 1 hybrid plasmids, and the other hybrid plasmid carrying ST107, ST395, ST20, and ST37 isolates were predominantly associated with a single healthcare institution (HCI). This pattern suggests a potential undetected plasmid outbreak and increases the possibility of horizontal gene transfer of these plasmids. This highlights the importance of long-read sequencing as available genomic data are somewhat limited and comparative plasmid analysis requires this approach. A more comprehensive understanding of the underlying evolutionary processes will therefore require expanded long-read sequencing.

An another further important observation related to plasmid structure involved the carbapenemase-encoding region. The *bla*_NDM-1_ and *bla*_NDM-5_ gene regions were in a conserved region on all identified *bla*_NDM_-carrying plasmids, bordered by the *IS26* and *ISSpu2* (*IS630*) transposases. Furthermore, in the immediately adjacent region, the *aph(3′)-VI* gene was bordered by *ISAba125* (*IS30* family) and *ISAba14* (*IS3 family*) insertion elements. The high similarity between the *bla*_NDM-1_ and *bla*_NDM-5_ regions suggests that *bla*_NDM-5_ likely evolved from *bla*_NDM-1_ through point mutations and that the IncF(K) plasmid may have acquired *bla*_NDM-5_ through multiple independent events. However, the close resemblance between the two gene regions may also reflect limitations of bioinformatics analysis as *bla*_NDM-5_ differs from *bla*_NDM-1_ by two amino acid substitutions (Val88Leu and Met154Leu). These observations suggest that IS26, a well-recognised mediator of antibiotic resistance gene mobilisation, plays a crucial role in the cointegration of this gene region into plasmids and in deletion events during plasmid evolution (Weber et al., 2019; Tóth et al., 2022; Guo et al., 2025; Zhou et al., 2025).

A marked difference was also observed between the two clusters regarding their siderophore repertoire. Yersiniabactin was absent from all isolates in Cluster 1 from the eastern region of the country, whereas it was detected in all Cluster 2 isolates originating from the capital and its surroundings. In these isolates, the yersiniabactin locus was associated with the three most common *ICEKp* elements (*ICEKp3, ICEKp4*, and I*CEKp12*), which can facilitate the dissemination of the ybt gene cluster. In addition to its role in iron acquisition, yersiniabactin can chelate other metal ions, including Cu^2+^, thereby contributing to protection against copper toxicity and redox-based phagocytic defence mechanisms. Although yersiniabactin is present in approximately one-third of clinical *K. pneumoniae* isolates, its loss appears relatively common, possibly because of the high metabolic cost associated with its biosynthesis (Lam et al., 2018; Jati et al., 2023; Lan et al., 2025). Moreover, yersiniabactin and salmochelin serve as important alternative iron acquisition systems when enterobactin is neutralised by the innate immune protein lipocalin-2. However, salmochelin-associated genes were not detected in any of our isolates.

Enterobactin, the highly conserved siderophore of *Klebsiella* spp., was detected in all isolates. Although the *entD* gene was absent in most strains, this finding has previously been reported in *bla*_OXA-48_-producing *K. pneumoniae* clinical isolates from the Netherlands and Spain (Jati et al., 2023).

Finally, aerobactin, one of the most important biomarkers of the hypervirulent pathotype, was detected in all isolates. This finding is consistent with international data showing that the complete aerobactin operon is present in more than 90% of hvKp strains and is considered a more specific marker of this pathotype than either enterobactin or yersiniabactin (Al Ismail et al., 2024).

However, defining the hypervirulent pathotype remains challenging, particularly lack of the universally accepted definition of hypervirulence. Consequently, reconstructing the evolutionary trajectories of *K. pneumoniae* and estimating the distribution of its pathotypes remain difficult because of inconsistencies in the available literature. In many studies, isolates carrying aerobactin and/or hypermucoidy-associated loci are classified as hypervirulent solely on the basis of these genetic markers, irrespective of their broader genetic background or evidence demonstrating a true hypervirulent phenotype *in vivo* (Tang et al., 2020; Wyres et al., 2020b; Jovicevic et al., 2026; Thacharodi et al., 2026). Moreover, hypervirulence is now considered a multifactorial trait determined by the combined action of multiple virulence-associated genes rather than by the presence of aerobactin and/or *rmpA* (Tang et al., 2020; Arcari and Carattoli, 2023). Meanwhile, our genomic analysis was complemented by the string test, which was historically regarded as a phenotypic marker of hypervirulence. However, subsequent studies demonstrated that a positive string test reflects hypermucoviscosity rather than hypervirulence itself. Not all hypervirulent strains exhibit a hypermucoviscous phenotype, whereas some hypermucoviscous isolates lack the genetic determinants associated with hypervirulence. Consistent with these observations, less than one quarter of the isolates in our collection displayed a hypermucoviscous phenotype (Russo et al., 2018; Wyres et al., 2020b; Arcari and Carattoli, 2023; Mai et al., 2023; Rao and Fernandes, 2025).

In summary, the convergence of hypervirulence and carbapenem resistance in *K. pneumoniae* has emerged as major global public health threats and may represent one of the most significant public health challenges of the 21st century, due to their association with increased morbidity and mortality worldwide. In Asia, the convergence has been frequently reported among multiple clonal lineages, including ST11, ST29, ST65, ST592, ST25, ST464, and ST23. Although ST23 remains the dominant lineage, the detection of emerging sequence types such as ST3507, ST3508, and ST3509 further highlights the increasing genetic diversity of hvKp populations (Thacharodi et al., 2026).

The European Centre for Disease Prevention and Control (ECDC) highlighted the emergence and spread of the globally dominant ST23-K1 CR-hvKP strains in Europe in its February 2024 alert. In contrast, our study highlights the significance of the spread of ST147 hv(a)CpKp strains in Hungary, consistent with observations reported from Italy and the United Kingdom (ECDC, 2024; Turton et al., 2024). Beyond Asia, the prevalence of hvKp and CR-hvKp has become increasingly global. In Qatar, ST231 and ST383 CR-hvKp strains with IncHI1B aerobactin and hypermucoidy-associated loci co-carrying plasmids have been detected. In Germany, two hvKp ST420 isolates were found to harbour chromosomally integrated virulence plasmids with enhanced siderophore production (Thacharodi et al., 2026). In Serbia, several CR-hvKp clones have been detected, including ST147, ST395, ST437, ST101, and ST11. Among these, *bla*_NDM-1_-producing ST147 strains were dominant, while the other clones were present only in small numbers (Jovicevic et al., 2026).

On the one hand, this suggests the spread of non-typical hvKp, classical MDR-Kp clones characterised by the acquisition of hva genes. On the other hand, in other regions, the acquisition of carbapenemase genes by typical hvKp clones appears to be more common. For example, in Ireland, a surveillance study detected CR-hvKp ST23 strains carrying *bla*_OXA-48_ together with major virulence loci. Similar typical hvKp strains harbouring both carbapenemase genes and major hypervirulence loci have also been reported in France, Italy, India, and Iran. Overall, the emergence of CR-hvKp reflects plasmid-mediated resistance and virulence convergence, its geographic expansion from East Asia to multiple continents, and the ongoing genetic diversification of hvKp populations (Thacharodi et al., 2026). Moreover, the convergence of hypervirulence and antimicrobial resistance is important from both the hypervirulence and antimicrobial resistance perspectives. The spread of carbapenemase genes leads to significant therapeutic limitations due to resistance to last-line carbapenems and is associated with poor clinical outcomes in severe infections, while hypervirulence increases pathogenicity and transmission potential (Zhu et al., 2021; Global Antimicrobial Resistance and Use Surveillance System (GLASS) Report 2022, 2022; Chen et al., 2023; Mendes et al., 2023). In addition, we identified deletions of the MgrB regulatory protein in 11 isolates, indicating potential colistin resistance (Bray et al., 2022), which was confirmed in most isolates, further narrowing the available treatment options.

Collectively, our findings and the international literature highlight a rapidly evolving molecular epidemiological landscape characterised by the increasing convergence of hypervirulence and multidrug resistance in *K. pneumoniae*. Our study highlights the significant genetic plasticity of ST147 strains and indicates that the convergence of hypervirulence and multidrug resistance is no longer limited to typical recognised hypervirulent clones. Instead, the acquisition of virulence determinants by multidrug-resistant lineages is becoming increasingly common across a wide range of *K. pneumoniae* clonal backgrounds. We observed the high prevalence of hva genes on IncHI1B/IncFIB(Mar) plasmids, underscoring the key role of these plasmids in the convergence of the two pathotypes. Our results also indicate the crucial role of transposases in gene mobilisation and the need for detailed analysis of plasmid diversity. However, our study is retrospective and limited to isolates submitted to the National Reference Laboratory and therefore may not completely reflect the national situation. Furthermore, the hypervirulent phenotype was not confirmed by *in vivo* assays and the study focused on the presence of hypervirulence-associated genetic determinants.

### 4.1 Limitations

A limitation of our study is that we examined only the virulence gene repertoire of the isolates, without conducting *in vivo* virulence assays; therefore, we consistently use the term “hypervirulence-associated” rather than hypervirulent. In addition, we focused on CpKp strains and investigated whether they had acquired hva genes. By contrast, an alternative approach could involve examining isolates derived from invasive infections, which might allow the identification of different types of convergent strains. The few *in vivo* studies available to date suggest that the virulence of convergent isolates is lower and that they exhibit fewer characteristics of the classical hypervirulent pathotype (Kochan et al., 2023; Duarte-Zambrano et al., 2026). However, because of the limited number of isolates studied, no definitive conclusions can be drawn from these observations. Accordingly, a systematic evaluation of clinical outcomes is also essential for assessing the clinical significance and risk associated with convergent isolates. Taken together, these findings highlight the need for an integrated classification system that takes into account genomic characteristics, *in vivo* virulence phenotype, and antibiotic resistance when characterising convergent *K. pneumoniae* isolates (Duarte-Zambrano et al., 2026).

## 5 Conclusion

Our study demonstrated the presence of hv(a)CpKp strains in Hungary during the study period. The most frequently detected clone was the ST147.

Furthermore, two evolutionary pathways of hv(a)CpKp convergence were observed within the ST147 clone: the acquisition of hybrid plasmids, and, in carbapenemase-producing ST147 lineages, the acquisition of plasmids carrying only hva genes. In the latter case, the high degree of plasmid similarity suggests vertical dissemination.

Overall, these findings indicate that two distinct ST147 lineages are circulating in Hungary and that classical MDR-Kp strains acquired hv genes via conjugative IncHI1B/IncFIB(Mar) plasmids.

These findings highlight the importance of routine molecular profiling of virulence determinants in *K. pneumoniae* isolates associated with severe invasive infections. Overall, our results emphasise the need for continuous genomic surveillance and plasmid-based epidemiological studies to better understand the dissemination of hv(a)Kp clones.

## Data Availability

The datasets presented in this study can be found in online repositories. The names of the repository/repositories and accession number(s) can be found at: https://www.ncbi.nlm.nih.gov/, PRJNA1405938.

## References

[ref1] Abdelsalam N. A. ElBanna S. A. Mouftah S. F. Cobo-Díaz J. F. Shata A. H. Shawky S. M. . (2024). Genomic dynamics of high-risk carbapenem-resistant *klebsiella pneumoniae* clones carrying hypervirulence determinants in Egyptian clinical settings. BMC Infect. Dis. 24:1193. doi: 10.1186/s12879-024-10056-1, 39438795 PMC11515790

[ref2] Al Ismail D. Campos-Madueno E. I. Donà V. Endimiani A. (2024). Hypervirulent *Klebsiella pneumoniae* (hvKp): overview, epidemiology, and laboratory detection. Pathog. Immun. 10, 80–119. doi: 10.20411/pai.v10i1.777, 39911145 PMC11792540

[ref5] Andrews S. (2010). FastQC: A Quality Control Tool for High Throughput Sequence Data. Babraham Bioinformatics. Available online at: https://www.bioinformatics.babraham.ac.uk/projects/fastqc/

[ref3] Arcari G. Carattoli A. (2023). Global spread and evolutionary convergence of multidrug-resistant and hypervirulent *Klebsiella pneumoniae* high-risk clones. Pathog. Glob. Health 117, 328–341. doi: 10.1080/20477724.2022.2121362, 36089853 PMC10177687

[ref4] Arena F. Menchinelli G. Di Pilato V. Torelli R. Antonelli A. Henrici De Angelis L. . (2022). Resistance and virulence features of hypermucoviscous *Klebsiella pneumoniae* from bloodstream infections: results of a nationwide Italian surveillance study. Front. Microbiol. 13:983294. doi: 10.3389/fmicb.2022.983294, 36204614 PMC9531727

[ref6] Bouras G. Grigson S. R. Papudeshi B. Mallawaarachchi V. Roach M. J. (2024). Dnaapler: a tool to reorient circular microbial genomes. J. Open Source Softw. 9:5968. doi: 10.21105/joss.05968

[ref7] Bray A. S. Smith R. D. Hudson A. W. Hernandez G. E. Young T. M. George H. E. . (2022). MgrB-dependent colistin resistance in *Klebsiella pneumoniae* is associated with an increase in host-to-host transmission. MBio 13:e03595–21. doi: 10.1128/mbio.03595-21, 35311534 PMC9040857

[ref8] Bulger J. MacDonald U. Olson R. Beanan J. Russo T. A. (2017). Metabolite transporter PEG344 is required for full virulence of hypervirulent *Klebsiella pneumoniae* strain hvKP1 after pulmonary but not subcutaneous challenge. Infect. Immun. 85:e00093–17. doi: 10.1128/IAI.00093-17, 28717029 PMC5607406

[ref9] Buzgó L. Kiss Z. Göbhardter D. Lesinszki V. Ungvári E. Rádai Z. . (2025). High prevalence of Cefiderocol resistance among New Delhi Metallo-β-lactamase producing *Klebsiella pneumoniae* high-risk clones in Hungary. Antibiotics 14, 475. doi: 10.3390/antibiotics14050475, 40426541 PMC12108422

[ref10] Carattoli A. Zankari E. Garciá-Fernández A. Larsen M. V. Lund O. Villa L. . (2014). In silico detection and typing of plasmids using plasmidfinder and plasmid multilocus sequence typing. Antimicrob. Agents Chemother. 58, 3895–3903. doi: 10.1128/AAC.02412-14, 24777092 PMC4068535

[ref11] Chen J. Zhang H. Liao X. (2023). Hypervirulent *Klebsiella pneumoniae*. Infect. Drug Resist. 16, 5243–5249. doi: 10.2147/IDR.S418523, 37589017 PMC10426436

[ref12] Chen L. Zheng D. Liu B. Yang J. Jin Q. (2016). VFDB 2016: hierarchical and refined dataset for big data analysis - 10 years on. Nucleic Acids Res. 44, D694–D697. doi: 10.1093/nar/gkv1239, 26578559 PMC4702877

[ref13] Chen S. Zhou Y. Chen Y. Gu J. (2018). Fastp: an ultra-fast all-in-one FASTQ preprocessor. Bioinformatics, 34, i884–i890. doi: 10.1093/bioinformatics/bty560 30423086 PMC6129281

[ref14] Choby J. E. Howard-Anderson J. Weiss D. S. (2020). Hypervirulent *Klebsiella pneumoniae* – clinical and molecular perspectives. J. Intern. Med. 287, 283–300. doi: 10.1111/joim.13007, 31677303 PMC7057273

[ref15] Damjanova I. Tóth Á. Pászti J. Hajbel-Vékony G. Jakab M. Berta J. . (2008). Expansion and countrywide dissemination of ST11, ST15 and ST147 ciprofloxacin-resistant CTX-M-15-type β-lactamase-producing *Klebsiella pneumoniae* epidemic clones in Hungary in 2005 - the new “MRSAs”? J. Antimicrob. Chemother. 62, 978–985. doi: 10.1093/jac/dkn287, 18667450

[ref16] De Coster W. Rademakers R. (2023). NanoPack2: population-scale evaluation of long-read sequencing data. Bioinformatics 39: btad311. doi: 10.1093/bioinformatics/btad311, 37171891 PMC10196664

[ref17] Drezen E. Rizk G. Chikhi R. Deltel C. Lemaitre C. Peterlongo P. . (2014). GATB: Genome Assembly & Analysis Tool box. Bioinformatics 30, 2959–2961. doi: 10.1093/bioinformatics/btu406, 24990603 PMC4184257

[ref18] Duarte-Zambrano L. Nava-Domínguez N. Mireles-Dávalos C. D. Becerril-Vargas E. González-Sánchez H. M. Rodríguez-Medina N. . (2026). Virulence and genomic features of hypervirulent *Klebsiella pneumoniae* species complex. Microb. Pathog. 213:108305. doi: 10.1016/j.micpath.2026.108305, 41581710

[ref19] Durrant M. G. Li M. M. Siranosian B. A. Montgomery S. B. Bhatt A. S. (2020). A Bioinformatic analysis of integrative Mobile genetic elements highlights their role in bacterial adaptation. Cell Host Microbe 27, 140–153.e9. doi: 10.1016/j.chom.2019.10.022, 31862382 PMC6952549

[ref20] European Centre for Disease Prevention and Control (2024). Emergence of hypervirulent Klebsiella pneumoniae ST23 carrying carbapenemase genes in EU/EEA countries, first update. 14 February 2024. ECDC: Stockholm. doi: 10.2900/993023

[ref21] EUCAST (2023). EUCAST disk diffusion method for antimicrobial susceptibility testing. Version 11.0. European Committee on Antimicrobial Susceptibility Testing. Available online at: https://www.eucast.org

[ref22] EUCAST (2024) EUCAST reading guide for broth microdilution Version 5.0. Available online at: https://www.eucast.org

[ref23] European Centre for Disease Prevention and Control (2025) Carbapenem-resistant Enterobacterales, third update –3 February 2025. ECDC: Stockholm. doi: 10.2900/8752612

[ref24] Falcone M. Tiseo G. Arcari G. Leonildi A. Giordano C. Tempini S. . (2022). Spread of hypervirulent multidrug-resistant ST147 *Klebsiella pneumoniae* in patients with severe COVID-19: an observational study from Italy, 2020-21. J. Antimicrob. Chemother. 77, 1140–1145. doi: 10.1093/jac/dkab495, 35040981 PMC9383231

[ref25] Freytag C. Timmer B. Kardos G. Laczkó L. (2024) P2244A comprehensive database for the direct identification of plasmid transfer genes. Available online at: https://escmid.reg.key4events.com/AbstractList.aspx?e=21&header=0&&ai=24221&preview=1&aig=1 (Accessed June 23, 2026).

[ref26] Frolova D. Lima L. Roberts L. Bohnenkämper L. Wittler R. Stoye J. . (2024). Applying rearrangement distances to enable plasmid epidemiology with pling. Microbial Genomics. 10:001300. doi: 10.1099/mgen.0.001300 39401066 PMC11472880

[ref27] Gilchrist C. L. M. Chooi Y. H. (2021). Clinker & clustermap.Js: automatic generation of gene cluster comparison figures. Bioinformatics 37, 2473–2475. doi: 10.1093/bioinformatics/btab007 33459763

[ref28] Global antimicrobial resistance and use surveillance system (GLASS) report 2022 (2022). Geneva: World Health Organization. Available online at: https://iris.who.int/bitstream/handle/10665/364996/9789240062702-eng.pdf?sequence=1 (Accessed October 28, 2024).

[ref29] gpsz/dep. (2024). Draw depth plot of bam. GitHub repository. Available online at: https://github.com/gpsz/dep. (Accessed November 18, 2024).

[ref30] Guo Z. Qin X. Yue M. Wu L. Li N. Su J. . (2025). IS26 carrying blaKPC−2 mediates carbapenem resistance heterogeneity in extensively drug-resistant *Klebsiella pneumoniae* isolated from clinical sites. Mob. DNA 16:13. doi: 10.1186/s13100-025-00351-2, 40128793 PMC11931797

[ref31] Gurevich A. Saveliev V. Vyahhi N. Tesler G. (2013). QUAST: quality assessment tool for genome assemblies. Bioinformatics 29, 1072–1075. doi: 10.1093/bioinformatics/btt086, 23422339 PMC3624806

[ref32] Han Y. L. Wen X. H. Zhao W. Cao X. S. Wen J. X. Wang J. R. . (2022). Epidemiological characteristics and molecular evolution mechanisms of carbapenem-resistant hypervirulent *Klebsiella pneumoniae*. Front. Microbiol. 13:1003783. doi: 10.3389/fmicb.2022.1003783, 36188002 PMC9524375

[ref33] Jati A. P. Sola-Campoy P. J. Bosch T. Schouls L. M. (2023). Widespread detection of yersiniabactin gene cluster and its encoding integrative conjugative elements (ICE Kp) among nonoutbreak OXA-48-producing *Klebsiella pneumoniae* clinical isolates from Spain and the Netherlands. Microbiol. Spectrum 11:4. doi: 10.1128/spectrum.04716-22, 37310221 PMC10434048

[ref34] Jia B. Raphenya A. R. Alcock B. Waglechner N. Guo P. Tsang K. K. . (2017). CARD 2017: expansion and model-centric curation of the comprehensive antibiotic resistance database. Nucleic Acids Res. 45, D566–D573. doi: 10.1093/nar/gkw1004, 27789705 PMC5210516

[ref35] Jolley K. A. Maiden M. C. J. (2010). BIGSdb: scalable analysis of bacterial genome variation at the population level. BMC Bioinformatics 11:595. doi: 10.1186/1471-2105-11-595, 21143983 PMC3004885

[ref36] Jovicevic M. Kabic J. Kekic D. Brankovic M. Zigmanovic A. S. Delic S. . (2026). The alarming rate of carbapenem-resistant *Klebsiella pneumoniae* in Serbia: a deep dive into genomic diversity, resistome, hypervirulence, and expansion of high-risk clone ST147/blaNDM-1. Microbiol. Res. 310:128543. doi: 10.1016/j.micres.2026.128543, 42119223

[ref37] Kochan T. J. Nozick S. H. Valdes A. Mitra S. D. Cheung B. H. Lebrun-Corbin M. . (2023). *Klebsiella pneumoniae* clinical isolates with features of both multidrug-resistance and hypervirulence have unexpectedly low virulence. Nat. Commun. 14:7962. doi: 10.1038/s41467-023-43802-1, 38042959 PMC10693551

[ref38] Kolmogorov M. Yuan J. Lin Y. Pevzner P. A. (2019). Assembly of long, error-prone reads using repeat graphs. Nat. Biotechnol. 37, 540–546. doi: 10.1038/s41587-019-0072-8, 30936562

[ref39] Lam M. M. C. Wick R. R. Judd L. M. Holt K. E. Wyres K. L. (2022). Kaptive 2.0: updated capsule and lipopolysaccharide locus typing for the *Klebsiella pneumoniae* species complex. Microb. Genom. 8:000800. doi: 10.1099/mgen.0.000800, 35311639 PMC9176290

[ref40] Lam M. M. C. Wick R. R. Watts S. C. Cerdeira L. T. Wyres K. L. Holt K. E. (2021). A genomic surveillance framework and genotyping tool for Klebsiella pneumoniae and its related species complex. Nat. Commun. 12:4188. doi: 10.1038/s41467-021-24448-3, 34234121 PMC8263825

[ref41] Lam M. M. C. Wick R. R. Wyres K. L. Gorrie C. L. Judd L. M. Jenney A. W. J. . (2018). Genetic diversity, mobilisation and spread of the yersiniabactin-encoding mobile element ICEKp in *Klebsiella pneumoniae* populations. Microb. Genom. 4:000196. doi: 10.1099/mgen.0.000196 PMC6202445 29985125

[ref42] Lan P. Lu Y. Fu Y. Yu Y. Zhou J. (2025). Siderophores and beyond: A comprehensive review of iron acquisition in *Klebsiella pneumoniae*. Virulence 16:2550621. doi: 10.1080/21505594.2025.2550621, 40899592 PMC12413041

[ref43] Letunic I. Bork P. (2024). Interactive tree of life (iTOL) v6: recent updates to the phylogenetic tree display and annotation tool. Nucleic Acids Res. 52, W78–W82. doi: 10.1093/NAR/GKAE268, 38613393 PMC11223838

[ref44] Liu C. Dong N. Chan E. W. C. Chen S. Zhang R. (2022). Molecular epidemiology of carbapenem-resistant *Klebsiella pneumoniae* in China, 2016–20. Lancet Infect. Dis. 22, 167–168. doi: 10.1016/S1473-3099(22)00009-3, 35092791

[ref45] Mai D. Wu A. Li R. Cai D. Tong H. Wang N. . (2023). Identification of hypervirulent *Klebsiella pneumoniae* based on biomarkers and galleria mellonella infection model. BMC Microbiol. 23:369. doi: 10.1186/s12866-023-03124-0, 38030994 PMC10685466

[ref46] Mendes G. Santos M. L. Ramalho J. F. Duarte A. Caneiras C. (2023). Virulence factors in carbapenem-resistant hypervirulent *Klebsiella pneumoniae*. Front. Microbiol. 14:1325077. doi: 10.3389/fmicb.2023.1325077, 38098668 PMC10720631

[ref47] Morgado S. Fonseca E. Vicente A. C. (2022). Genomics of *Klebsiella pneumoniae* species complex reveals the circulation of high-risk multidrug-resistant pandemic clones in human, animal, and environmental sources. Microorganisms 10:2281. doi: 10.3390/microorganisms10112281, 36422351 PMC9697336

[ref48] Nguyen L. T. Schmidt H. A. Von Haeseler A. Minh B. Q. (2015). IQ-TREE: a fast and effective stochastic algorithm for estimating maximum-likelihood phylogenies. Mol. Biol. Evol. 32, 268–274. doi: 10.1093/molbev/msu300, 25371430 PMC4271533

[ref49] Oxford Nanopore Technologies Ltd. (2018) nanoporetech/medaka: Sequence correction provided by ONT Research. Available online at: https://github.com/nanoporetech/medaka (Accessed December 10, 2025).

[ref50] Page A. J. Taylor B. Delaney A. J. Soares J. Seemann T. Keane J. A. . (2016). SNP-sites: rapid efficient extraction of SNPs from multi-FASTA alignments. Microb. Genom. 2:e000056. doi: 10.1099/mgen.0.000056, 28348851 PMC5320690

[ref51] Parks D. H. Imelfort M. Skennerton C. T. Hugenholtz P. Tyson G. W. (2015). CheckM: assessing the quality of microbial genomes recovered from isolates, single cells, and metagenomes. Genome Res. 25, 1043–1055. doi: 10.1101/gr.186072.114, 25977477 PMC4484387

[ref52] Peirano G. Chen L. Kreiswirth B. N. Pitout J. D. D. (2020) Emerging antimicrobial-resistant high-risk *Klebsiella pneumoniae* clones ST307 and ST147. Antimicrob Agents Chemother. 64:e01148–20. doi: 10.1128/AAC.01148-20 32747358 PMC7508593

[ref53] Prjibelski A. Antipov D. Meleshko D. Lapidus A. Korobeynikov A. (2020). Using SPAdes de novo assembler. Curr. Protoc. Bioinformatics 70:1. doi: 10.1002/cpbi.102, 32559359

[ref54] Rao S. S. Fernandes A. M. (2025). Detection of Hypervirulence genes and drug resistance in *Klebsiella pneumoniae* in diagnostic microbiology. Scientifica (Cairo). 2025:8545710. doi: 10.1155/sci5/8545710, 40787605 PMC12335906

[ref55] Raro O. H. F. Nordmann P. Pino M. D. Findlay J. Poirel L. (2023). Emergence of carbapenemase-producing hypervirulent *Klebsiella pneumoniae* in Switzerland. Antimicrob. Agents Chemother. 67:3. doi: 10.1128/aac.01424-22, 36853006 PMC10019205

[ref56] Robertson J. Nash J. H. E. (2018). MOB-suite: software tools for clustering, reconstruction and typing of plasmids from draft assemblies. Microb. Genom. 4:000206. doi: 10.1099/mgen.0.000206, 30052170 PMC6159552

[ref57] Russo T. A. Marr C. M. (2019). Hypervirulent *Klebsiella pneumoniae*. Available online at: https://journals.asm.org/journal/cmr (Accessed May 15, 2019)

[ref58] Russo T. A. Nordmann P. Dominguez Pino M. (2018). Identification of biomarkers for differentiation of hypervirulent *Klebsiella pneumoniae* from classical *K. pneumoniae*. J. Clin. Microbiol. 56:e00776–18. doi: 10.1128/JCM.00959-18 29925642 PMC6113484

[ref59] Santajit S. Indrawattana N. (2016). Mechanisms of antimicrobial resistance in ESKAPE pathogens. Biomed. Res. Int. 2016:2475067 doi: 10.1155/2016/2475067 27274985 PMC4871955

[ref60] Schwengers O. Barth P. Falgenhauer L. Hain T. Chakraborty T. Goesmann A. (2020). Platon: identification and characterization of bacterial plasmid contigs in short-read draft assemblies exploiting protein sequence-based replicon distribution scores. Microb. Genom. 6, 1–12. doi: 10.1099/mgen.0.000398, 32579097 PMC7660248

[ref61] Seemann T. (2014). Prokka: rapid prokaryotic genome annotation. Bioinformatics 30, 2068–2069. doi: 10.1093/bioinformatics/btu153, 24642063

[ref62] Seemann Torsten (2026a). Abricate. Available online at: https://github.com/tseemann/abricate (Accessed December 10, 2025).

[ref63] Seemann Torsten (2026b). mlst. Available online at: https://github.com/tseemann/mlst (Accessed December 10, 2025).

[ref64] Shapovalova V. V. Chulkova P. S. Ageevets V. A. Nurmukanova V. Verentsova I. V. Girina A. A. . (2024). High-risk lineages of hybrid plasmids carrying virulence and Carbapenemase genes. Antibiotics 13:1224. doi: 10.3390/antibiotics13121224, 39766615 PMC11726917

[ref65] Shimoyama Yuki (2024). pyGenomeViz: A genome visualization python package for comparative genomics. Available online at: https://github.com/moshi4/pyGenomeViz (Accessed December 10, 2025).

[ref66] Shoja S. Ansari M. Bengar S. Rafiei A. Shamseddin J. Alizade H. (2022) Bacteriological characteristics of hypervirulent *Klebsiella pneumoniae* rmpA gene (hvKp-rmpA)-harboring strains in the south of Iran. Available online at: http://ijm.tums.ac.ir 10.18502/ijm.v14i4.10233 PMC9867641 36721517

[ref67] Simão F. A. Waterhouse R. M. Ioannidis P. Kriventseva E. V. Zdobnov E. M. (2015). BUSCO: assessing genome assembly and annotation completeness with single-copy orthologs. Bioinformatics 31, 3210–3212. doi: 10.1093/bioinformatics/btv351, 26059717

[ref68] Spadar A. Perdigão J. Campino S. Clark T. G. (2023). Large-scale genomic analysis of global *Klebsiella pneumoniae* plasmids reveals multiple simultaneous clusters of carbapenem-resistant hypervirulent strains. Genome Med. 15:3. doi: 10.1186/s13073-023-01153-y, 36658655 PMC9850321

[ref69] Stanton T. D. Hetland M. A. K. Löhr I. H. Holt K. E. Wyres K. L. (2025). Fast and accurate in silico antigen typing with Kaptive 3. Microbial Genomics. 11:001428 doi: 10.1099/mgen.0.001428 40553506 PMC12188004

[ref70] Sun Z. Zhang J. Wang C. Chen J. Li P. Su J. . (2025). The pivotal role of IncFIB(mar) plasmid in the emergence and spread of hypervirulent carbapenem-resistant *Klebsiella pneumoniae*. Science Advances. 11:eado9097. doi: 10.1126/sciadv.ado9097 39888998 PMC11784837

[ref71] Tang M. Kong X. Hao J. Liu J. (2020). Epidemiological characteristics and formation mechanisms of multidrug-resistant Hypervirulent *Klebsiella pneumoniae*. Front. Microbiol. 11:581543. doi: 10.3389/fmicb.2020.581543, 33329444 PMC7714786

[ref72] Tavoschi L. Forni S. Porretta A. Righi L. Pieralli F. Menichetti F. . (2020). Prolonged outbreak of New Delhi metallo-betalactamase-producing carbapenem-resistant Enterobacterales (NDM-CRE), Tuscany, Italy, 2018 to 2019. Eurosurveillance 25:2000085. doi: 10.2807/1560-7917.ES.2020.25.6.2000085, 32070467 PMC7029447

[ref73] Thacharodi A. Ahmed T. Hassan S. Singh P. Pugazhendhi A. (2026). Carbapenem resistance and hypervirulence in *Klebsiella pneumoniae* are raising global public health concerns in the 21st century. iScience 29:114782. doi: 10.1016/j.isci.2026.114782, 41767277 PMC12936834

[ref74] Tonkin-Hill G. MacAlasdair N. Ruis C. Weimann A. Horesh G. Lees J. A. . (2020). Producing polished prokaryotic pangenomes with the Panaroo pipeline. Genome Biol. 21:180. doi: 10.1186/s13059-020-02090-4, 32698896 PMC7376924

[ref75] Tóth K. Tóth Á. Kamotsay K. Németh V. Szabó D. (2022). Population snapshot of the extended-spectrum β-lactamase-producing *Escherichia coli* invasive strains isolated from a Hungarian hospital. Ann. Clin. Microbiol. Antimicrob. 21:3. doi: 10.1186/s12941-022-00493-8, 35144632 PMC8829994

[ref76] Turton J. F. Perry C. McGowan K. Turton J. A. Hope R. (2024). *Klebsiella pneumoniae* sequence type 147: a high-risk clone increasingly associated with plasmids carrying both resistance and virulence elements. J. Med. Microbiol. 73:001823. doi: 10.1099/jmm.0.001823, 38629482 PMC11084618

[ref77] Tzouvelekis L. S. Markogiannakis A. Psichogiou M. Tassios P. T. Daikos G. L. (2012). Carbapenemases in Klebsiella pneumoniae and other Enterobacteriaceae: an evolving crisis of global dimensions. Clin. Microbiol. Rev. 25, 682–707. doi: 10.1128/CMR.05035-11, 23034326 PMC3485753

[ref78] Vaser R. Sović I. Nagarajan N. Šikić M. (2017). Fast and accurate de novo genome assembly from long uncorrected reads. Genome Res. 27, 737–746. doi: 10.1101/gr.214270.116, 28100585 PMC5411768

[ref79] Weber R. E. Pietsch M. Frühauf A. Pfeifer Y. Martin M. Luft D. . (2019). IS26-mediated transfer of blaNDM–1 as the Main route of resistance transmission during a polyclonal, multispecies outbreak in a German hospital. Front. Microbiol. 10:2817. doi: 10.3389/fmicb.2019.02817, 31921015 PMC6929489

[ref80] Wood D. E. Lu J. Langmead B. (2019). Improved metagenomic analysis with kraken 2. Genome Biol. 20:257. doi: 10.1186/s13059-019-1891-0, 31779668 PMC6883579

[ref81] Wyres K. L. Lam M. M. C. Holt K. E. (2020a). Population genomics of *Klebsiella pneumoniae*. Nat. Rev. Microbiol. 18, 344–359. doi: 10.1038/s41579-019-0315-1, 32055025

[ref82] Wyres K. L. Nguyen T. N. T. Lam M. M. C. Judd L. M. Van Vinh Chau N. Dance D. A. B. . (2020b). Genomic surveillance for hypervirulence and multi-drug resistance in invasive *Klebsiella pneumoniae* from south and Southeast Asia. Genome Med. 12:11. doi: 10.1186/s13073-019-0706-y, 31948471 PMC6966826

[ref83] Wyres K. L. Wick R. R. Gorrie C. Jenney A. Follador R. Thomson N. R. . (2016). Identification of Klebsiella capsule synthesis loci from whole genome data. Microb. Genom. 2:e000102. doi: 10.1099/mgen.0.000102, 28348840 PMC5359410

[ref84] Wyres K. L. Wick R. R. Judd L. M. Froumine R. Tokolyi A. Gorrie C. L. . (2019). Distinct evolutionary dynamics of horizontal gene transfer in drug resistant and virulent clones of *Klebsiella pneumoniae*. PLoS Genet. 15:e1008114. doi: 10.1371/journal.pgen.1008114, 30986243 PMC6483277

[ref85] Ye M. Tu J. Jiang J. Bi Y. You W. Zhang Y. . (2016). Clinical and genomic analysis of liver abscess-causing *Klebsiella pneumoniae* identifies new liver abscess-associated virulence genes. Front. Cell. Infect. Microbiol. 6:165. doi: 10.3389/fcimb.2016.00165, 27965935 PMC5126061

[ref86] Zhou J. Meng X. Ni S. Yang Y. Zhang Q. Wu L. . (2025). IS26-mediated cointegration generates a plasmid co-harbouring blaIMP-4 and blaKPC-2 in *Klebsiella pneumoniae*. J. Glob. Antimicrob. Resist. 42, 61–65. doi: 10.1016/j.jgar.2025.02.009, 39971271

[ref87] Zhu J. Wang T. Chen L. Du H. (2021). Virulence factors in Hypervirulent *Klebsiella pneumoniae*. Front. Microbiol. 12:642484. doi: 10.3389/fmicb.2021.642484, 33897652 PMC8060575

